# Dual Ni/Co-hemin metal–organic framework-PrGO for high-performance asymmetric hybrid supercapacitor

**DOI:** 10.1038/s41598-023-39553-0

**Published:** 2023-08-01

**Authors:** Kimia Zarean Mousaabadi, Ali A. Ensafi, Erfan Naghsh, Jin-Song Hu, Behzad Rezaei

**Affiliations:** 1 Department of Chemistry, Isfahan University of Technology, Isfahan, 84156-83111 Iran; 2 Department of Chemistry and Biochemistry, University of Arkansas, Fayetteville, AR 72701 USA; 3 Department of Electrical, Computer and Biomedical Engineering, Toronto Metropolitan University, Toronto, Canada; 4 Institute of Chemistry, Chinese Academy of Sciences, Beijing, 100190 China

**Keywords:** Energy science and technology, Materials science, Nanoscience and technology

## Abstract

In this study, we conducted direct synthesis of a dual metal–organic framework (Ni/Co-Hemin MOF) on phosphorous-doped reduced graphene oxide (PrGO) to serve as an active material in high-performance asymmetrical supercapacitors. The nanocomposite was utilized as an active material in supercapacitors, exhibiting a noteworthy specific capacitance of 963 C g^−1^ at 1.0 A g^−1^, along with a high rate capability of 68.3% upon increasing the current density by 20 times, and superior cycling stability. Our comprehensive characterization and control experiments indicated that the improved performance can be attributed to the combined effect of the dual MOF and the presence of phosphorous, influencing the battery-type supercapacitor behavior of GO. Additionally, we fabricated an asymmetric hybrid supercapacitor (AHSC) using Ni/Co-Hemin/PrGO/Nickel foam (NF) and activated carbon (AC)/NF. This AHSC demonstrated a specific capacitance of 281 C g^−1^ at 1.0 A g^−1^, an operating voltage of 1.80 V, an impressive energy density of 70.3 Wh kg^−1^ at a high power density of 0.9 kW kg^−1^. Notably, three AHSC devices connected in series successfully powered a clock for approximately 42 min. These findings highlight the potential application of Hemin-based MOFs in advanced supercapacitor systems.

## Introduction

Energy is considered the most critical scientific subject in the twenty-first century^1,2^. To survive the earth, renewable energy is essential to reducing greenhouse gas emissions and air pollution^3^. Hence, new energy generation technologies like solar^4^, wind^5^, and fuel cells^6^ require devices to store energy. Li-ion batteries and supercapacitors are the two main electrical energy storage systems. They developed over the years for portable devices as well as smart grid deployments^7^. Supercapacitors can store a large amount of charge compared to conventional capacitors, deliver energy quickly, have fast charging ability, have a long lifetime, offer superior low-temperature performance, are eco-friendly, and have low costs. Moreover, unlike batteries, they do not explode even if it is overcharged^8–11^.

On the other hand, Ragone plot^12^ illustrates supercapacitors' importance in their high specific power density. Besides, the capacitance in supercapacitors is influenced by equivalent series resistance and electrode and electrolyte materials and affects the operating voltage^13,14^. Therefore, for the best performance of a supercapacitor, it needs to have high capacitance, high operating voltage, and low resistance^15^. From these, electrode material, among all parameters, plays an essential role in developing the supercapacitor performance^15^. In other words, Hybrid supercapacitor devices are crucial for the advancement of electrochemical energy storage systems that can offer high energy storage capacity at a low operational cost^16^.

One of these electrode materials that appears effective is MOFs, porous hybrid materials consisting of metal ions or metal clusters coordinated to organic linkers^17–19^. Structures of this type offer the following advantages: high internal surface area, high porosity, structural and chemical tunability, and good stability. Besides, MOFs can be controlled in terms of porosity due to pore uniformity and atomic-level structure, dimension, geometry, functionality, and flexibility in network topology^20,21^. But, most pristine MOFs are poor conductors^22,23^. To overcome this deficiency, one of the most common strategies is to combine MOFs with carbon materials (reduced graphene oxide and carbon nanotubes) or conductive polymers (polypyrrole and polyaniline)^24^. Additionally, the incorporation of reduced graphene oxide (rGO) in composites can serve as an effective means of impeding the aggregation and restacking of graphene, both during the fabrication process and in practical usage^25^. The exceptional mechanical and chemical durability exhibited by rGO makes it an excellent scaffold material for the active component, which can effectively mitigate structural degradation and thereby enhance the cyclic stability of the system^26^.

This research synthesized dual MOFs/ PrGO as an active material. In other words, carbon-based materials such as GO have been extensively studied and utilized in industrial production for capacitors and batteries. The advantages of GO for use in supercapacitors are abundance, non-toxicity, facile manufacturing, low cost, high chemical stability, good electronic and mechanical properties, high specific surface area, and a vast working temperature range^27–29^. However, the performance of GO in supercapacitors is limited due to nanosheet stacking and agglomeration. This problem exists because of the van der Waals forces and *π–π* interactions, therefore, it dramatically decreases the specific capacitance^30^. To put it more simply, macroscopic properties are affected by the electrode material microstructure^31^. Some researchers are used different methods, such as synthesis GO with porous 3D architectures in the form of hydrogels, organic gels, and foams to provide a larger surface area and better electron transport channels^30,32^. The other method is modifying GO, including chemical doping with different heteroatoms (F, B, S, N, and P), which provide capacitive behavior and reduce the aggregation of GO sheets^33,34^. Among these heteroatoms, phosphorus great much attention for doping GO because the electronegativity of P (2.19) is lower than C (2.55), and the atomic radius size of P (195 pm) is more than C (170 pm), which causes polarization and distortion in the graphene lattice followed by the specific capacitance, surface area, and electrochemical performance were improved^35,36^. In other words, when phosphorus is doped in GO, P electrons contribute to the graphene *π*-system to increase charge carriers^30^.

This study uses MOFs with hemin ligands for supercapacitor applications for the first time. Hemin, an iron-containing porphyrin with chlorine, is widely used to synthesize electrocatalysts for fabricating electrochemical sensors^37,38^ and energy production, such as hydrogen and oxygen evolution reactions^39,40^. Besides, porphyrin-base structures such as Hemin have several advantages for utilizing active materials in supercapacitors. These structures have the following benefits: non-toxicity, low cost, and highly exposed active site in the active substance due to its highly conjugated structure (M–N_4_)^41,42^. Therefore, we tried synthesizing dual Ni/Co-Hemin MOF (NCH) and compositing with PrGO through the one-pot co-synthesis method to increase their cycle life, specific surface area, and pseudo-capacitance behavior. This method causes the uniform distribution of Ni-MOF and Co-MOF particles, and PrGO acts as a conductive bridge. Afterward, various spectroscopic and microscopic methods studied the structure of the obtained nanocomposite. The electrochemical behavior of this nanocomposite was first investigated in a three-electrode system for supercapacitor applications. The results of this study revealed that NCH/PrGO nanocomposite is a promising active material as an AHSC with wide operating voltage and acceptable value of energy density versus several related materials (Fig. S1).

## Results and discussion

### Characterization

**Figure 1:**
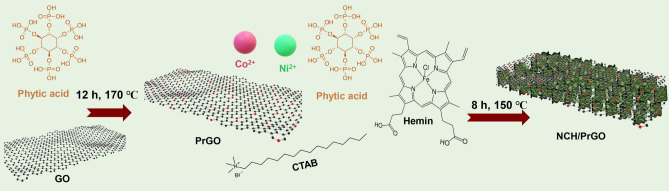
Schematic illustration of the synthesis of NCH/PrGO nanocomposite.

First, GO was doped with P using a facile hydrothermal method that used phytic acid as a phosphorous source to obtain PrGO hydrogel. Afterward, the novel NCH MOF was grown on graphene sheets. Moreover, phytic acid and CTAB are used as nucleating and kinetic control agents, respectively (Fig. 1).

**Figure 2:**
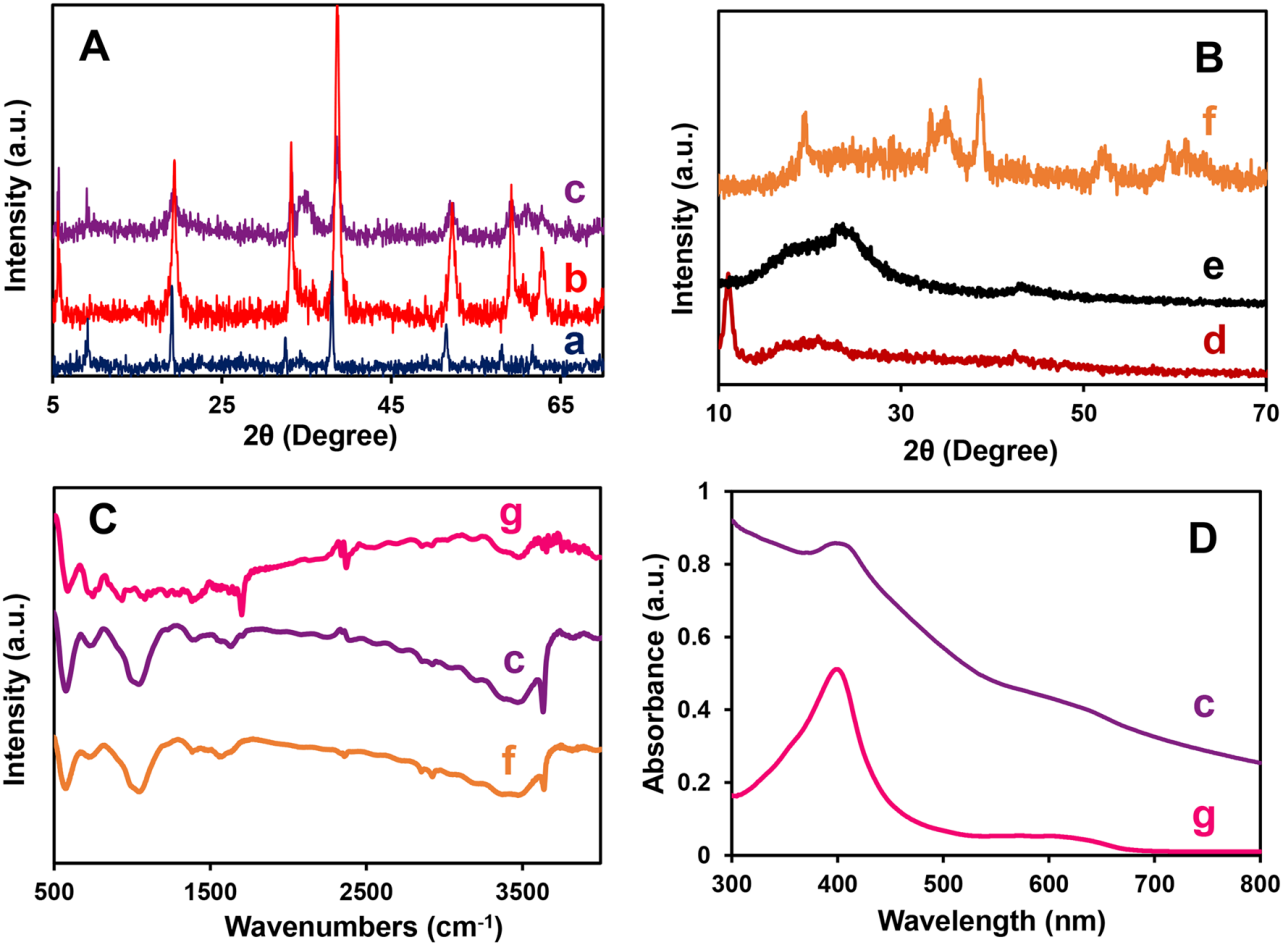
(**A**,**B**) XRD, (**C**) FT-IR, and (**D**) UV–Vis spectra of (a) CH, (b) NH, (c) NCH, (d) GO, (e) PrGO, (f) NCH/PrGO, and (g) Hemin.

Accordingly, X-ray diffraction (XRD) was used to characterize the structure of GO, PrGO, NH, CH, NCH, and NCH/PrGO (Fig. 2A,B). In the GO pattern, the peak at 2θ = 10.8° is attributed to the (001) plane that revealed the oxidation of graphite to form GO^43^, while in the PrGO pattern, the appearance of two broad peaks at 23.1° (002) and 43.8° (101) and the disappearance of the peak at 10.8° indicated that GO was reduced successfully^30^. Additionally, the broadening peaks of PrGO are due to a few layers of stacked graphene sheets forming the material's framework due to the poor stacking direction of graphene sheets^32^. As can be seen, in the NCH pattern, the main diffraction peaks at 5.8°, 9.1°, 19.9°, 33.7°, 36°, 39.1°, 52.8°, 59.7°, 61.3°, and 63.4° suggesting the crystalline structure of this MOF. Moreover, by comparing the XRD patterns of NCH with CH and NH, the diffraction peaks of both MOFs that appear in dual MOF can be concluded. According to the XRD pattern obtained from NCH/PrGO, its index peaks are equal to the patterns of dual MOF and PrGO, so their presence of them in the final nanocomposite is confirmed. Many attempts were done to obtain single crystal, but due to the very low solubility of this structure in most solvents, attempts were unsuccessful.

Fourier-transform infrared spectroscopy (FT-IR) spectra of Hemin, NCH, and NCH/PrGO are illustrated in Fig. 2C. In all spectra, the bands at 3451, 2927, and 1604 cm^−1^ are attributed to the O–H, C–H, and C=N/C=C of the protoporphyrin (IX) ring system^44^. The band's intensity at 1703 cm^−1^ was attributed to the C=O stretching vibration that decreased in the NCH spectrum, compared with the Hemin spectrum. Furthermore, 1465, 1392, and 1004 cm^−1^ absorption bands are corresponded to the B3u vibration of porphyrin, –CH_3_ of Hemin, and =C–H deformation vibration of the olefin. All results confirm the successful coordination of the metals and –COOH groups^37,45,46^. Besides, spectrum f reveals the NCH/PrGO is well composite. The soret (~ 400 nm) and Q (~ 630 nm) bands of Hemin appeared in the UV–Vis spectrum of NCH (Fig. 2D). Consequently, these results prove the MOF structure was successfully formed. Moreover, ICP-OES results reveal the molar ratio of elements in the NCH structure. Co, Ni, and Fe weight percent equal 32.6, 10.4, and 1.2%, respectively.

**Figure 3:**
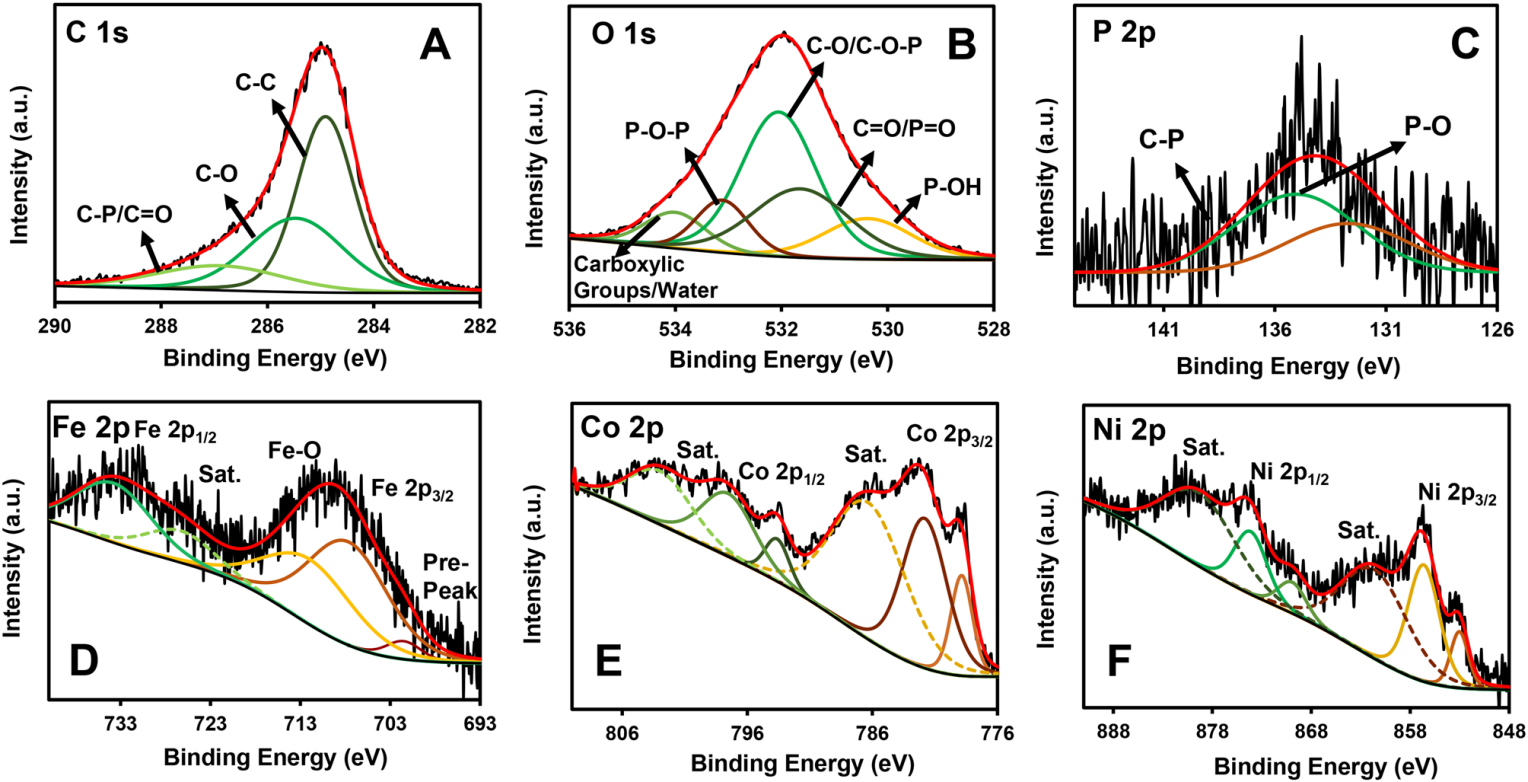
(**A**–**F**) High-resolution spectra of C 1s, O 1s, P 2p, Fe 2p, Co 2p, and Ni 2p, respectively, of NCH/PrGO.

To characterize the chemical state of the bonded element and the phase composition of NCH/PrGO, X-ray photoelectron spectroscopy (XPS) measurements were carried out. Figure 3A–F illustrates the high-resolution spectra of C 1s, O 1s, P 2p, Fe 2p, Co 2p, and Ni 2p, respectively. C 1s spectrum shows peaks at 285.01 (C–C bond), 285.7 (C–O bond), and 287.1 (C=O, C–P bonds) eV in PrGO^47^. The O 1s peaks at 529.8, 531.7, 532.9, 533.2, and 534.9 eV are associated with the P–OH, C=O/P=O, C–O/C–O–P, P–O–P, and water/carboxylic groups respectively^48^. The high-resolution P 2p XPS spectrum showed two peaks at 133.2 (C–P bond) and 134.3 eV (P–O bond)^48^. Moreover, the P–O peak intensity is higher than the P–C peak intensity revealing the GOs and phytic acids oxygen atoms led to the partial oxidation of P–C bonding in the thermal treatment^30^. Therefore, the results indicated that P had been successfully incorporated into the graphene lattices. It was stated in the introduction that reversible redox reactions in the P–O and C–P groups increase the overall energy density and, consequently, improve the electrochemical performance. The peaks at 700.1 (Fe pre-peak), 708.3 (Fe 2p_3/2_), 713 (Fe–O), and 734.1 (Fe 2p_1/2_) eV in a high-resolution scan of the Fe 2p region are related to Fe^3+^ in Hemin^49^. The main peaks appeared in the high-resolution spectrum of the Co 2P attributed to Co 2p_3/2_ and Co 2p_1/2_, which indicates the existence^50^ of Co^2+^ and Co^3+^. Likewise, the Ni 2p_3/2_ and Ni 2p_1/2_ spectra demonstrated four peaks that contribute to Ni^2+^ (related to Ni–O octahedral bonding) and Ni^3+^ in the nanocomposite^45^.

**Figure 4:**
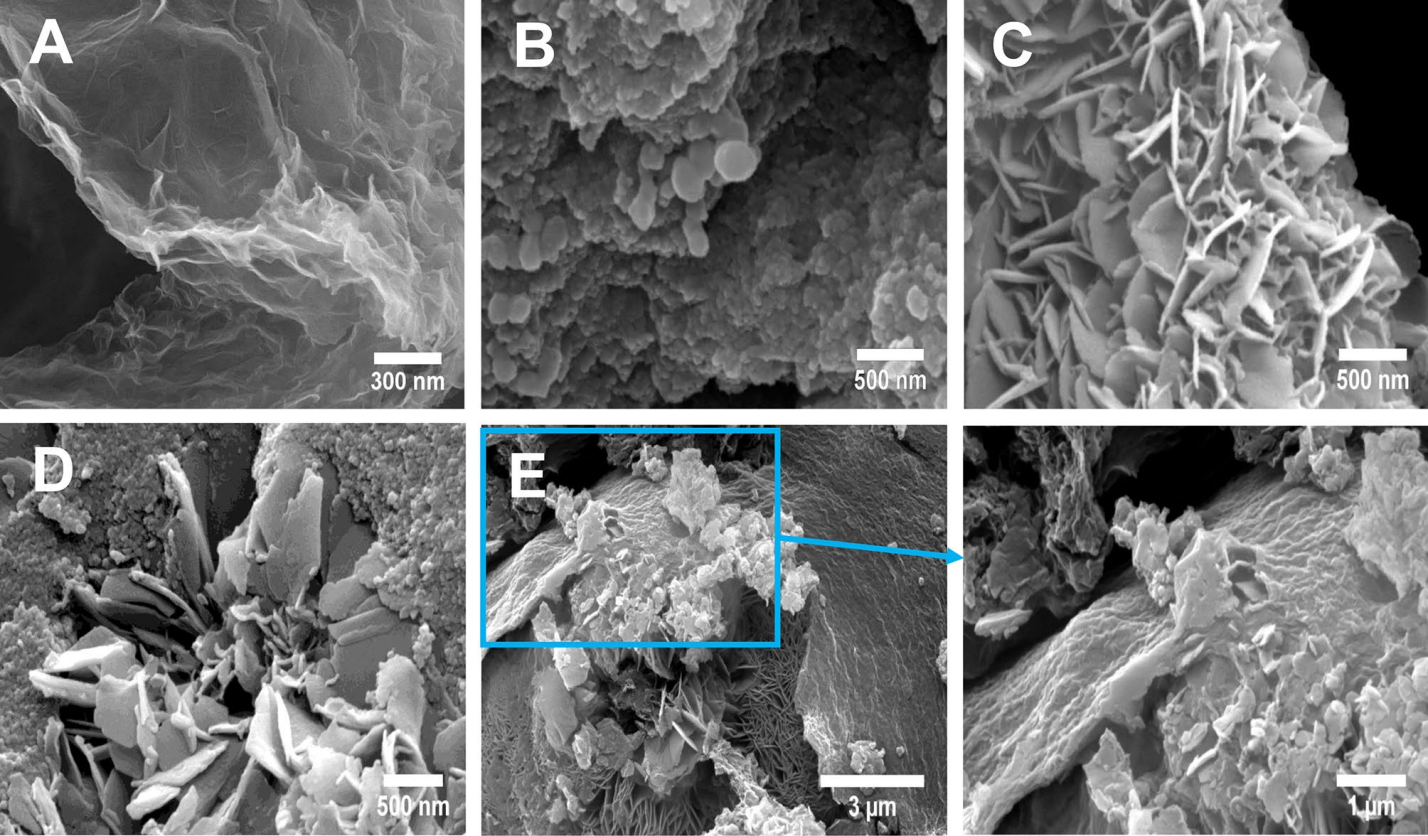
FE-SEM images of (**A**) PrGO, (**B**) CH, (**C**) NH, (**D**) NCH, and (**E**) NCH/PrGO.

Microscopy images were used for the investigation of the morphology. Figure 4A–E illustrates field-emission scanning electron microscopy (FE-SEM) images of (A) PrGO, (B) CH, (C) NH, (D) NCH, and (E) NCH/PrGO. PrGO image displays 3D porous network and GO sheets with an effective surface area to improve the fast diffusion of the ions^51^. The morphology of CH is illustrated in Fig. 4B. Moreover, the NH MOF image showed a wrinkled nanoflakes shape with an average thickness of about 29 nm. On the other hand, the FE-SEM image of the nanocomposite proved both MOF structures that have been successfully composited with the GO sheets. Additionally, NCH MOF structures may provide an extensive contact area between the electrolyte and the electrode due to having a large specific surface area. Therefore, the path of OH^−^ diffusion is reduced, and the nanocomposite has sufficient space to diminish deformation and improve the cycle life during charge and discharge.

The high-resolution TEM (HR-TEM) image of NCH MOF is shown in Fig. S2A–C revealing that this structure is well-crystallized and has uniformity. Moreover, the TEM image (Fig. S2D) of NCH/PrGO is consistent with the FE-SEM images. Energy-dispersive X-ray spectroscopy (EDS) analysis and elemental mapping were used to study the nanocomposite structure elements and their distribution. The EDS analysis of NCH/PrGO attests to the composition formation and the presence of the P element, which shows the successful doping of GO with this heteroatom. The results obtained from Ni, Co, and Fe (Hemin) mapping show a uniform distribution in NCH. Moreover, the elemental mapping of C and P provide information about the uniform composition of NCH and PrGO (Fig. S3).

According to the results obtained from the reported analysis, it can be concluded that the NCH MOF is successfully synthesized, and the NCH/PrGO nanocomposite is formed.

### Electrochemical performance of the NCH/PrGO

The electrochemical performance of the different electrodes was investigated by cyclic voltammetry (CV), galvanostatic charge/discharge (GCD), and electrochemical impedance spectroscopy (EIS) in 1.0 M KOH solution in a three-electrode system.

Figure S4A illustrated CV curves of the NCH/PrGO/NF electrodes with different amounts of PrGO (0.2, 0.4, 0.6, and 0.8 g L^−1^) at a scan rate of 20 mV s^−1^ in a potential window from 0.00 to 0.80 V vs. Hg/HgO. With increasing the amount of PrGO up to 0.6 g L^−1^, the area of the CV curve was increased; after this amount, the area dramatically decreased. In addition, this claim was proven by the Brunauer–Emmett–Teller (BET) data. As a result, the specific surface area of CH, NH, NCH, and NCH/PrGO was measured to be 40.21, 68.41, 71.44, and 92.54 m^2^ g^−1^, respectively. According to these observations, increasing the amount of P improves the capacitive behavior of the nanomaterial because of the lattice distortions of GO. Still, over-doping with P may damage graphene's original structure and reduce its capacitance and conductivity^30^. Moreover, as shown in Fig. S4B, the results of the GCD curves confirm the same trend; NCH/PrGO_0.6_ revealed the highest capacitance (963 C g^−1^), and this electrode was used for subsequent investigation. On the other hand, comparing their FE-SEM images gives us similar results with electrochemical data. In the FE-SEM image of NCH/PrGO_0.2_, due to the low amount of PrGO, we cannot see the PrGO sheets, and in the FE-SEM image of NCH/PrGO_0.8_, PrGO sheets cover the porosity of dual MOF structures (Fig. S5).

In mathematics, computer science, and chemistry, an optimization process is a problem of finding the best value of a parameter of all possible solutions^52^. In fact, in optimization problems, we are looking for the largest or smallest value that a function can take. Many of these problems can be solved by finding the appropriate cost function and then using optimization problem-solving techniques to find the maximum or minimum required value of the respective cost function. Hence, the effect of amounts of PrGO on the capacitance behavior is optimized with MATLAB R2008b. According to the numbers obtained from the practical experiments, the cost function was estimated as follows:

$$y = - 15667x^{3} + 20412x^{2} - 7550.8x + 1529$$

Figure S6 shows the estimated cost function. The next step is the optimal point to obtain the maximum capacitance. To obtain the optimal point, we must take the cost function mentioned in Eq. (1) of the first-order derivative in terms of the amounts of PrGO and set it to zero.

1 $$\frac{dy}{{dx}} = - 47001x^{2} + 40820x - 7550.8 = 0$$

$$x_{opt}^{(1)} = 0.26\quad and\quad x_{opt}^{(2)} = 0.6$$

The two optimal points (0.6 and 0.26) are obtained by zeroing the first-order derivative of the cost function. According to the cost function estimated at 0.6, the maximum capacity is obtained, which is very consistent with the value obtained from practical experiments.

**Figure 5:**
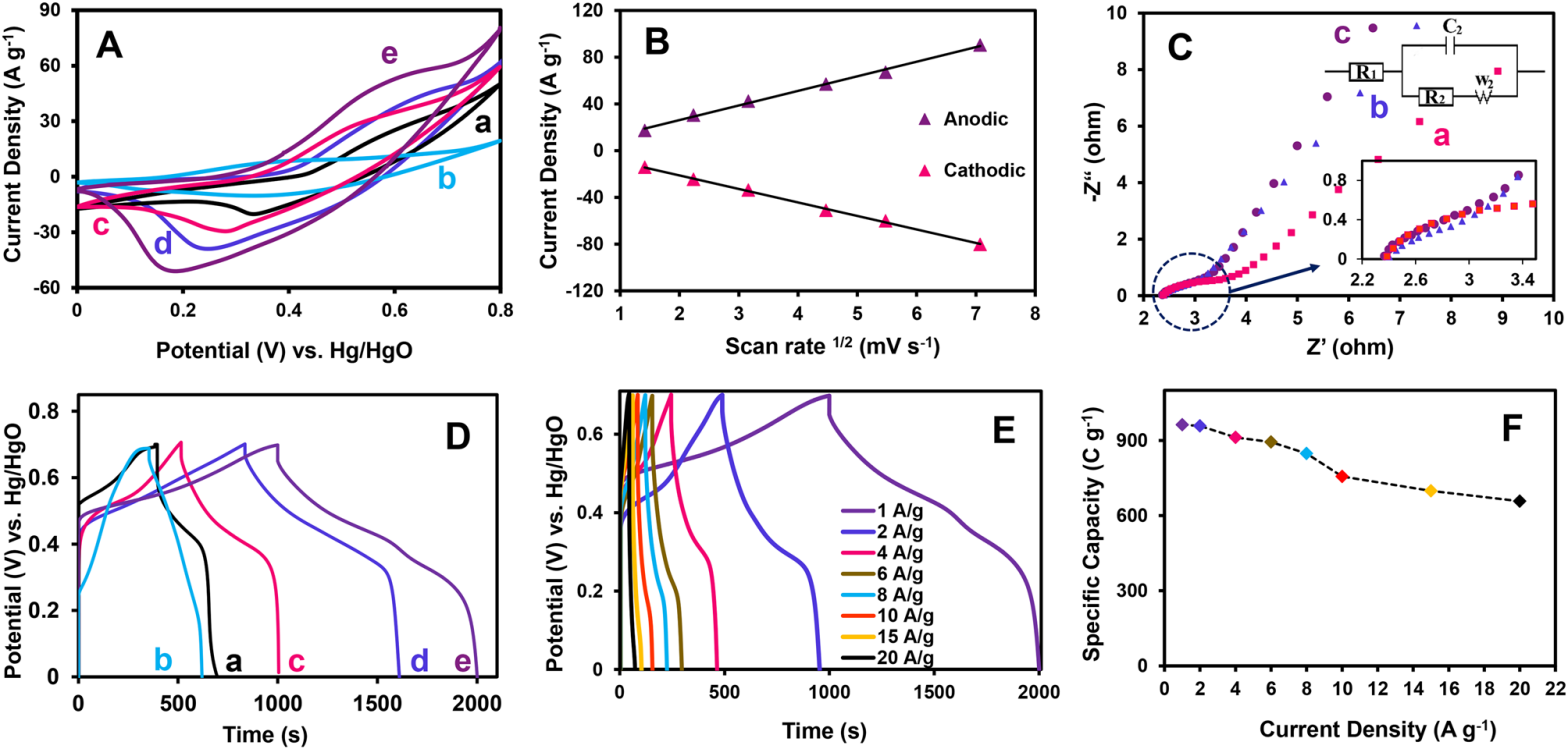
Electrochemical performance measurements in a three-electrode system. (**A**) CV curves of (a) NH/NF, (b) CH/NF, (c) NCH/NF, (d) NCH/rGO/NF, and (e) NCH/PrGO/NF at a scan rate of 20 mV s^−1^ in the potential window from 0.00 to 0.80 V vs. Hg/HgO, (**B**) The relationships between current densities and the square root of scan rates of NCH/PrGO/NF, (**C**) Nyquist plots of the (a) NCH/NF, (b) NCH/rGO/NF, and (c) NCH/PrGO/NF electrodes from 100 kHz to 100 mHz at OCP (inset: equivalent circuit), (**D**) GCD curves of the (a) NH/NF, (b) CH/NF, (c) NCH/NF, (d) NCH/rGO/NF, and (e) NCH/PrGO/NF electrodes at a current density of 1 A g^−1^, (**E**) GCD curves of the NCH/PrGO/NF electrode at different current densities, and (**F**) the relevance of the specific capacity of the NCH/PrGO/NF electrode with current densities in 1.0 M KOH.

Figure 5A shows the comparative CV curves of (a) NH/NF, (b) CH/NF, (c) NCH/NF, (d) NCH/rGO/NF, and (e) NCH/PrGO/NF at a scan rate of 20 mV s^−1^ in the potential window from 0.0 to 0.80 V vs. Hg/HgO. The NCH/PrGO/NF CV showed the highest current density, revealing excellent faradic behavior and a larger specific capacity. The results showed that a dual MOF announced superior performance than a monometallic MOF due to the synergic effects of the metals. The peaks in the NCH/PrGO/NF CV curve are related to the overlaid peaks of two pairs of the redox peaks of cobalt and nickel. Moreover, GO plays an important role in enhancing the electrochemical properties because of increasing the surface area and conductivity. P, as a dopant, increased the charge density (the types of P groups, especially C–P and P–O groups, are responsible for augmenting the overall energy density through reversible redox reactions)^53^ and caused structural distortions. Unlike the conventional methods, such as chemical vapor deposition, which produces hazardous gaseous for doping GO, the technique used in the present study for doping GO is safe. Additionally, phytic acid is a natural and green P source compared with other sources.

The peak currents enhanced by increasing the scan rates due to the diffusion of OH^−^ ions. The shape of the CVs shows the quasi-reversible reaction. Additionally, the cathodic peak potential was shifted to a negative potential, while the anodic peak potential was moved to a positive value (Fig. S7A). As a result, Low reversible Faradic processes occur because of the active material's internal diffusion resistance at a high scan rate. Figure 5B illustrates the relationships between the current densities and the square root of the scan rates that show OH^−^ diffusion. Augmenting scan rates enhances the CV integrated area but decreases the charge storage because of the difficulty of OH^−^ into the electrode internal structures and pores and poor interaction between the electrode material and the electrolyte. Figure S7B depicts the areal capacitance of NCH/PrGO. The Dunn method was utilized to examine the comprehensive performance of charge storage, encompassing both capacitive and diffusion-controlled processes^54^. Through the investigation of capacitive and diffusion-controlled charge storage properties at different scan rates, as depicted in Fig. S8, it was determined that capacitive charge storage prevails at high scan rates, whereas diffusion-controlled charge storage predominates at low scan rates. This trend illustrates that at scan rates of 2 and 50 mV s^−1^, the contribution of capacitive charge storage accounts for 24% and 87% of the total stored charge, respectively.

EIS is a perfect method to better study the charge-transfer behavior, interfacial properties, and capacitive properties of the electrodes. The Nyquist plot of the NCH/NF, NCH/rGO/NF, and NCH/PrGO/NF electrode from 100 kHz to 100 mHz frequency range, with an equivalent circuit (inset) plot for fitting the EIS data of NCH/PrGO/NF electrode at open circuit potential (OCP) is shown in Fig. 5C. The diameter of the semicircle part in the high-frequency region (that is related to the charge transfer resistance (R_ct_) at the interface between the electrode surface/electrolyte) of NCH/PrGO/NF displays a lower R_ct_ (1.05 Ω) than NCH/NF (1.16 Ω), and NCH/rGO/NF (2.05 Ω). Moreover, In the low-frequency region, the Warburg resistance (R_w_) varies as a function of the ion diffusion resistance of the electrolyte inside the electrode materials; the steep slope of the NCH/PrGO/NF curve shows the lower diffusive resistance and the pure capacitor behavior. Therefore, this electrode revealed fast electron transfer kinetics, lower internal resistance, and good conductivity.

The GCD curve of the NCH/PrGO/NF delivers the highest capacitance (963 C g^−1^) among other electrodes due to more active sites and high conductivity (Fig. 5D). Besides, the GCD curve of the NCH/PrGO/NF electrode at different current densities (Fig. 5E); the specific capacities were 963, 956, 912, 894, 847, 756, 699, and 657 C g^−1^ at 1, 2, 4, 6, 8, 10, 15, and 20 A g^−1^, respectively. Thus, the specific capacity of the electrodes gradually decreased with increasing the current density due to diffusing the ions slowly through the electrolyte during the GCD process. When the current density is increased 20 times, the specific capacitance of this electrode retains a 68.3% initial value (Fig. 5F). This result reveals a high-rate capability for NCH/PrGO/NF.

Cycle stability is one of the essential characteristics of evaluating supercapacitors. Figure S9 depicts that 86.63% of the initial specific capacitance of NCH/PrGO/NF is maintained after 3000 successive GCDs performed at a current density of 10 A g^−1^. The result confirms its desirable stability, but a reduction in the specific capacitance resulted from the destruction of NCH/PrGO and a loss of its active sites. Figure S10A displays the impedance spectra and FESEM images of NCH/PrGO/NF before and after cycling. The resistance of the electrolyte and interface exhibited a modest increase from 1.05 to 2.64 Ω after 3000 cycles. This change in resistance is attributed to the maintained structural integrity of the cross-linked structure and the favorable stability of the film formed in situ at the electrode–electrolyte interface during prolonged cycling. Moreover, FESEM image of the NCH/PrGO/NF electrode after 3000 cycles reveals instances where the structure exhibits cohesion, resulting in reduced ion diffusion during GCD cycles. This observation is illustrated in Fig. S10B.

**Figure 6:**
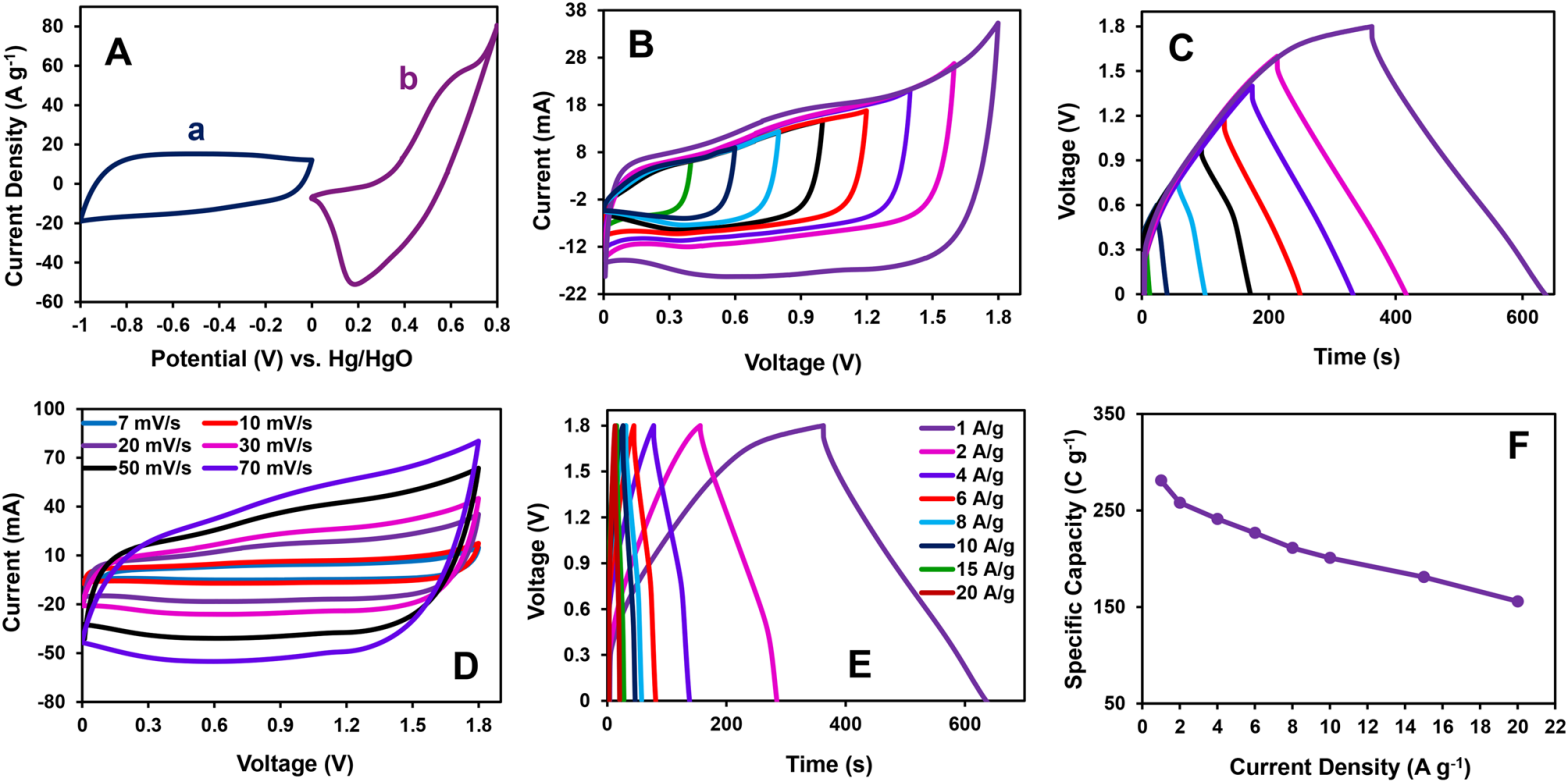
Electrochemical performance measurements in AHSC device (**A**) CV curves of the AC/NF (a) and NCH/PrGO/NF (b) electrodes at a scan rate of 20 mV s^−1^ in a three-electrode system in 1.0 M KOH, (**B**) CV curves at different potential windows (0.40–1.80 V) at a scan rate of 20 mV s^−1^, (**C**) GCD curves at different potential windows (0.40–1.80 V) at a current density of 1.0 A g^−1^, (**D**) CV curves at different scan rate (7–70 mV s^−1^), (**E**) GCD curves at different current densities (1–20 A g^−1^), and (**F**) the relevance of the specific capacity with current densities of the NCH/PrGO/NF//AC/NF AHSC.

A significant shortcoming of supercapacitors is their low energy density and limited by the voltage window. The asymmetric hybrid supercapacitor (AHSC) was fabricated to improve the voltage window; this configuration consists of two different kinds of electrodes. The NCH/PrGO/NF//AC/NF was assembled as an AHSC device to evaluate energy and power density performance. The NCH/PrGO/NF and AC/NF were applied as positive and negative electrodes, respectively. Figure 6A depicts the CV curves of the NCH/PrGO/NF and AC/NF at the scan rate of 20 mV s^−1^ in the three-electrode system for activation. CV (at scan rates of 20 mV s^−1^) and GCD (at 1.0 A g^−1^) curves of NCH/PrGO/NF//AC/NF AHSC device in different potential windows are illustrated in Fig. 6B,C. Since the limitation associated with oxygen evolution reaction at a voltage higher than 1.80 V, the operating voltage of the NCH/PrGO/NF//AC AHSC device was extended to 1.80 V. Having a wide operating voltage allows this ASC device to be used in practical applications.

Afterward, the CVs were performed at different scan rates (7–70 mV s^−1^) (Fig. 6D). Consequently, both faradic behavior from NCH/PrGO/NF and electric double-layer capacitance from AC was confirmed for the charge storage. Moreover, as displayed in Fig. 6E (the GCD curves of the NCH/PrGO/NF//AC/NF AHSC device at various current densities), the shape of the GCE curves is consistent with the CV curves in a term of charge storage. The specific capacitance of the NCH/PrGO/NF//AC/NF AHSC device is calculated as 281, 257, 248, 230, 211, 201, 181, and 156 C g^−1^ at 1, 2, 4, 6, 8, 10, 15, and 20 A g^−1^, respectively. Hereupon, 55.5% of the initial specific capacitance retains after increasing the current density 20 times (Fig. 6F). When the current densities increase due to the limited diffusion effect, the active material will be less utilized, and the faradic process will be limited. Moreover, the carbon-based electrode has a higher ion exchange rate than a pseudocapacitive electrode, which causes a charge imbalance between the electrodes. Therefore, the specific capacitances of the AHSC device decrease gently. This result reveals the excellent rate capability for NCH/PrGO/NF//AC/NF AHSC devices.

**Figure 7:**
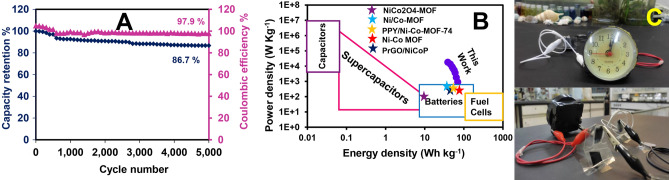
(**A**) Cycling performance and coulombic efficiency of the NCH/PrGO/NF//AC/NF AHSC at 10 A g^−1^, (**B**) Ragone plot of the NCH/PrGO/NF//AC/NF AHSC^55–59^, and (**C**) the clock run by two all-solid-state NCH/PrGO/NF//AC/NF AHSCs connected in series.

The long-term stability and coulombic efficiencies of the NCH/PrGO/NF//AC/NF AHSC device after 5000 successive GCD cycles at 10 A g^−1^ are shown in Fig. 7A. The specific capacitance and coulombic efficiency of the NCH/PrGO/NF//AC/NF AHSC device after 5000 GCD cycles decrease by about 13.3% and 2.1% of its initial value, respectively. To further investigate the long-term stability of NCH/PrGO/NF//AC/NF AHSC, a floating test was performed for 100 h. The potential of AHSC was maintained at a current density of 5 A g^−1^ at the potential of 1.75 V for 10 h and repeated ten times. For every 10 h, 5 GCD test was performed, and capacity retention of 93.1% was maintained after 120 h (Fig. S11). Hence, NCH/PrGO/NF//AC/NF AHSC displays superb electrochemical stability.

The Ragone plot of the NCH/PrGO/NF//AC/NF AHSC device shows a maximum energy density of 70.3 Wh kg^−1^ at 0.9 kW kg^−1^, and the energy density remains at 38.9 Wh kg^−1^ at 18 kW kg^−1^ (Fig. 7B). Afterward, three NCH/PrGO/NF//AC/NF AHSC devices are connected in series to investigate a practical application. This device successfully ran the alarm clock (1.5 V) for 42 min (Video 1 and Fig. 7C). The comparison between the NCH/PrGO as an active material for supercapacitors and some previous work in three-electrode and two-electrode systems reveal superior performance (Table 1).

**Table 1:** A comparison between this work and previous work based on PrGO or Ni-Co MOFs.

Active material/positive electrode	Media	Three-electrode system	Two-electrode system		
		Specific capacitance/capacity	Negative electrode	Energy density (Wh kg^−1^)	Power density (W kg^−1^)
Ni/Co MOF^60^	KOH 2.0 M	339.3 C g^−1^ at 1.0 A g^−1^	–	–	–
PrGO/NiCoP^55^	KOH 3.0 M	2586.2 F g^−1^ at 1.0 A g^−1^	AC	49.7	366
P-doped graphene^30^	H_2_SO_4_ 1.0 M	388.5 F g^−1^ at 1.0 mA cm^−2^	–	–	–
Phosphorus-doped graphene aerogel^61^	Na_2_SO_4_ 1.0 M	225.3 F g^−1^ at 1.0 A g^−1^	–	–	–
Ni/Co-MOF^56^	KOH 2.0 M	568 C g^−1^ at 1.0 A g^−1^	rGO	42.24	800
NiCo_2_O_4_-MOF^57^	KOH 3.0 M	531 F g^−1^ at 1.0 A g^−1^	AC	9.4	82
Ni-Co MOF^58^	KOH 1.0 M	833 C g^−1^ at 0.5 A g^−1^	AC	77.7	450
q-2D-MOF/rGO film^62^	H_2_SO_4_ 1.0 M	292.5 F g^−1^ at 0.7 A g^−1^	–	–	–
PPY/Ni-Co-MOF-74^59^	KOH 6.0 M	849 C g^−1^ at 1.0 A g^−1^	AC	58.4	747.6
NCH/PrGO (this study)	KOH 1.0 M	963 C g^−1^ at 1.0 A g^−1^	AC	70.3	900

## Methods

### Synthesis of PrGO

Please see the supporting information.

### Synthesis of NCH/PrGO

First, 0.2, 0.4, 0.6, and 0.8 mg mL^−1^ uniform suspension of PrGO was prepared. Then, 0.64 mmol Ni(NO_3_)_2_·6H_2_O and 0.32 mmol Co(NO_3_)_2_·6H_2_O were added to 20 mL of each suspension and ultrasonic for 1 h. After that, 0.068 phytic acids and 0.15 mmol cetrimonium bromide (CTAB) were added to the solution and stirred for 30 min (solution A). Besides, 20 mL (18 mL distilled water + 2.0 mL ammonium hydroxide solution) of 0.50 mM Hemin solution was prepared (solution B). Afterward, solution B was gradually added to solution A and stirred for 15 min. The resulting solution was transferred into a 70 mL Teflon-lined stainless-steel autoclave, kept at 150 °C for 8 h, cooled naturally to room temperature, and washed with distilled water several times. Finally, the dark slate grey sediments (NCH/PrGO_0.2_, NCH/PrGO_0.4_, NCH/PrGO_0.6_, and NCH/PrGO_0.8_) were collected and dried at 60 °C overnight.

Apparatus and methods are described in the supporting information.

## Conclusion

To summarize, novel Ni/Co-Hemin MOF/PrGO nanocomposite was synthesized as an active material for a high-performance hybrid supercapacitor. Afterward, this nanocomposite was characterized by different spectroscopic (XRD, XPS, and FT-IR) and microscopic (FE-SEM and TEM) techniques. This nanocomposite demonstrates excellent electrochemical properties, including high specific capacity 963 C g^−1^ at 1.0 A g^−1^ (68.3%) retained after increasing the current density 20 times), and good cycling life. The advantages of the composition of MOF with carbon-based material such as GO that is doped with heteroatom are (1) high conductivity, (2) non-toxicity, (3) high porosity, (4) high internal and specific surface area, (5) facile synthesis, (6) good stability, (7) chemical and structural tunability, (8) superior mechanical and electronic properties, (9) low cost, (10) enhance charge carriers, (11) increase pseudo-capacitance property, (12) synergistic effect of bimetallic and (13) prevent agglomeration. Moreover, Hemin is a promising ligand for synthesizing MOF that is used in supercapacitor applications for the first time and has some advantages, such as low cost, non-toxic, and highly conjugated structure. Furthermore, the AHSC was assembled on Ni/Co-Hemin MOF/PrGO/NF (as the positive electrode) and AC (as the negative electrode), resulting in a specific capacity of 281 C g^−1^ with a high energy density of 70.3 Wh kg^−1^ and power density of 0.9 kW kg^−1^. The excellent electrochemical performance confirms Ni/Co-Hemin MOF/PrGO's potential as an active material for AHSCs for future energy storage systems used in electronic devices and vehicles. Finally, this work aims to show the perspective of MOF-based Hemin (a natural product) for energy storage systems.

## Supplementary Information

Supplementary Information.

Supplementary Video 1.

## Data Availability

The datasets supporting the conclusions of this article are included within the article.
