# Supplementary material for: Dual Ni/Co-hemin metal–organic framework-PrGO for high-performance asymmetric hybrid supercapacitor
Source: Sci Rep. 2023 Aug 1;13:12422. doi: 10.1038/s41598-023-39553-0 (PMC10393980; doi:10.1038/s41598-023-39553-0)
Supplement: Supplementary file 1 — Supplementary Information. [file 41598_2023_39553_MOESM1_ESM.docx]

**Supporting Information for**

Dual Ni/Co-Hemin Metal-Organic Framework-PrGO for High-Performance Asymmetric Hybrid Supercapacitor

Kimia Zarean Mousaabadi, Ali A. Ensafi *, Erfan Naghsh, Jin-Song Hu, and Behzad Rezaei

* Department of Chemistry, Isfahan University of Technology, Isfahan 84156-83111, IRAN. E-mail: Ensafi@iut.ac.ir; aensafi@uark.edu, aaensafi@gmail.com.


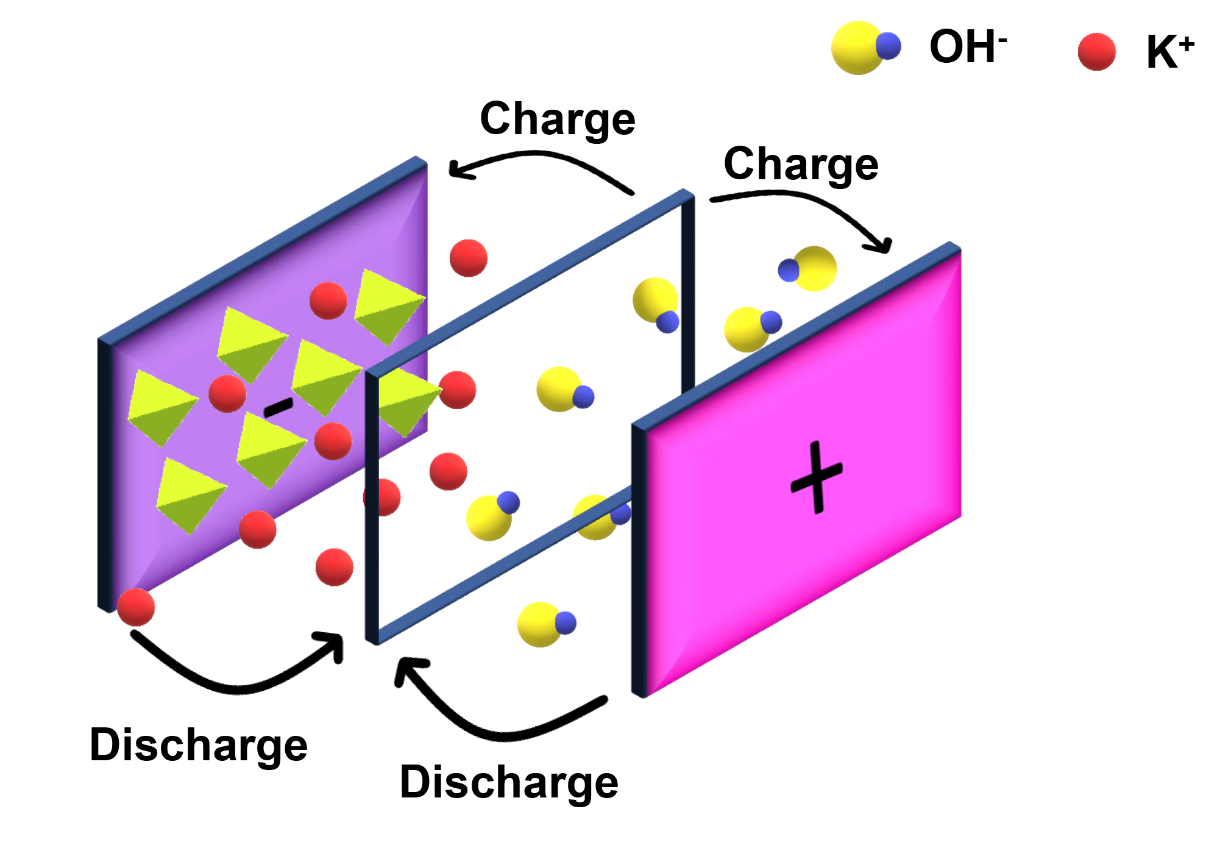


**Figure S1** The configuration of the HSCs


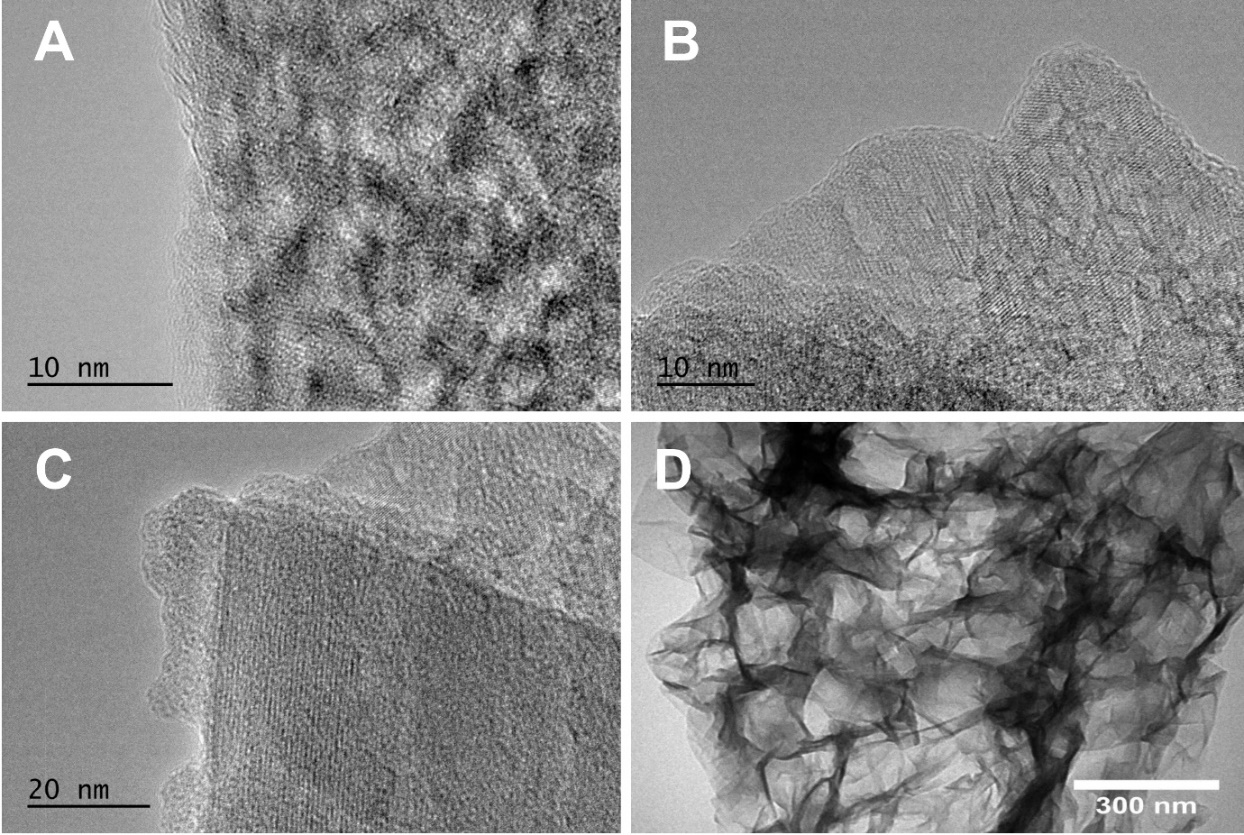


**Figure S2 (A-C)** HR-TEM images of NCH and **(D)** TEM image of NCH/PrGO


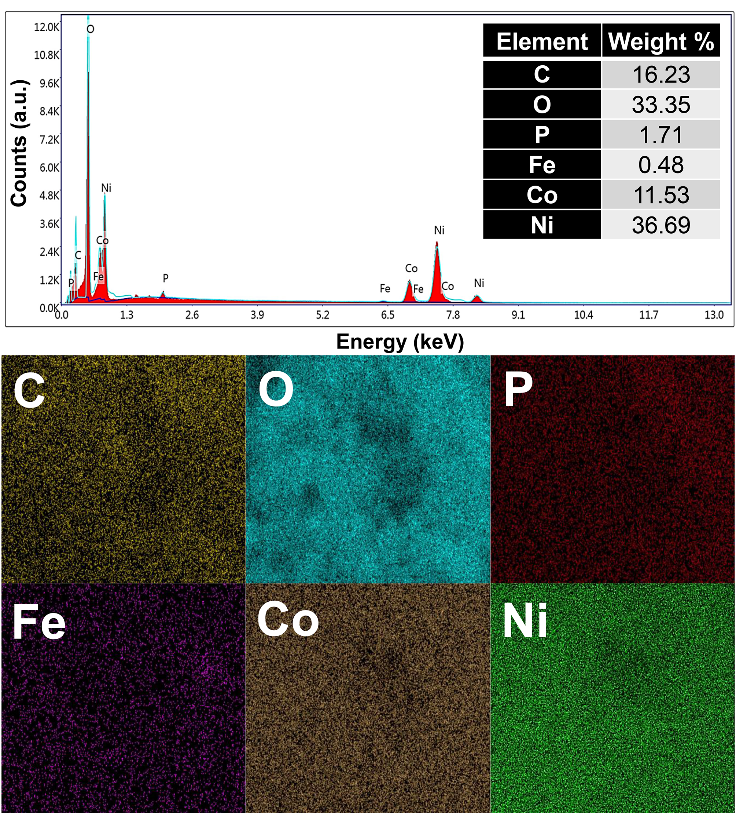


**Figure S3** EDS and Elemental mapping of NCH/PrGO.


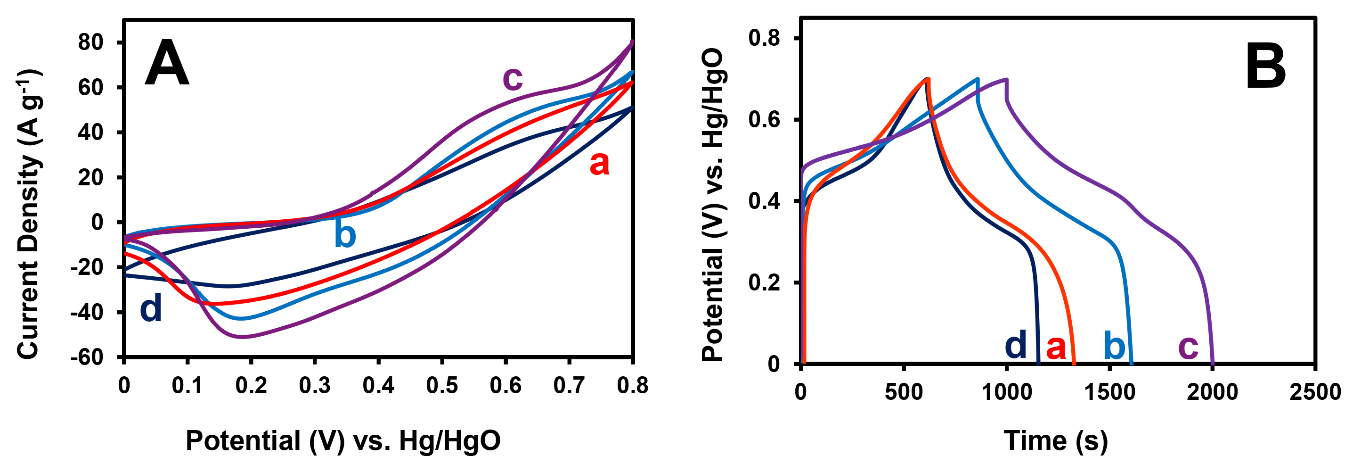


**Figure S4** **(A)** CV and **(B)** GCD curves of the NCH/PrGO/NF electrodes with different amounts of PrGO (a) 0.2, (b) 0.4, (c) 0.6, and (d) 0.8 g L^-1^ at a scan rate of 20 mV s^-1^ in KOH 1.0 M.


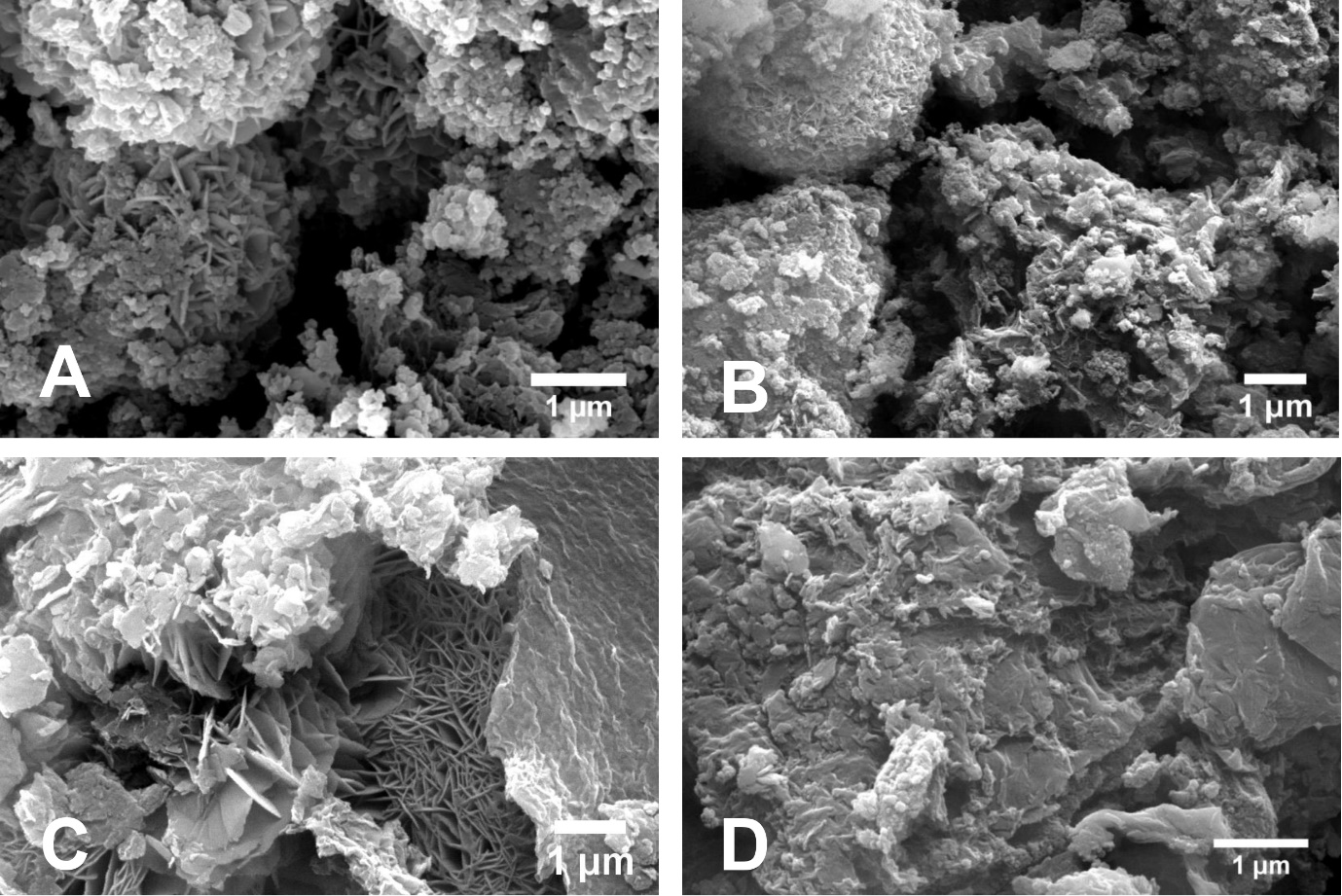


**Figure S5** FE-SEM images of the NCH/PrGO with different amounts of PrGO **(A)** 0.2, **(B)** 0.4, **(C)** 0.6, and **(D)** 0.8 g L^-1^.


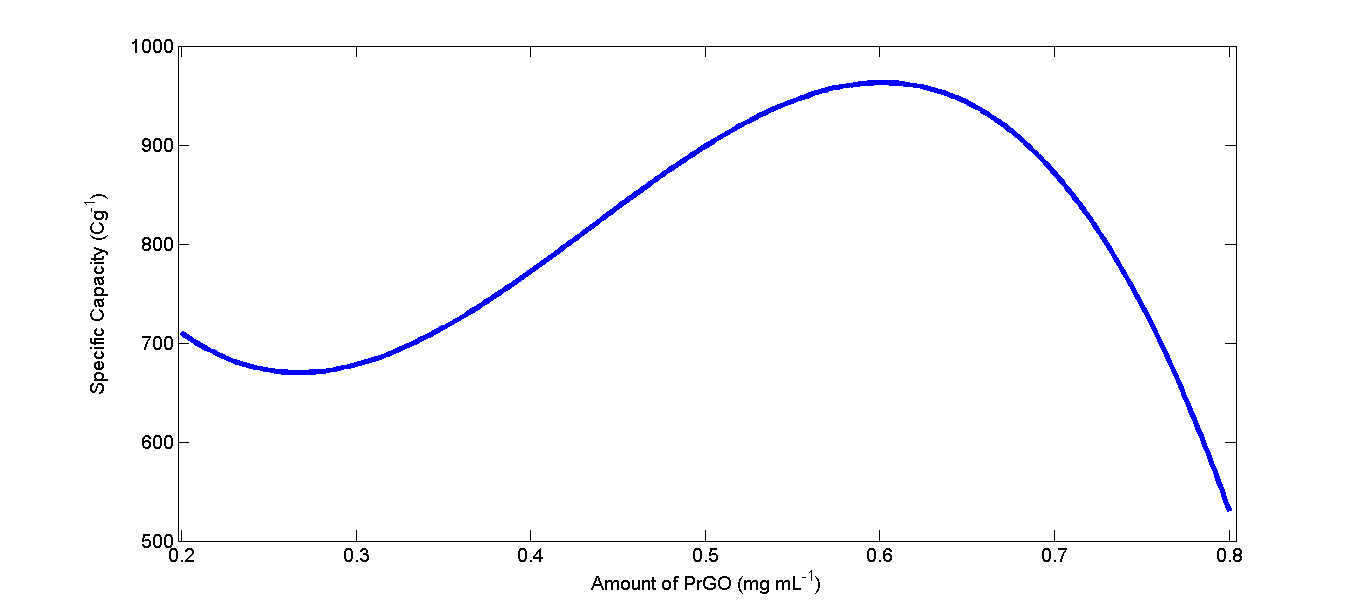


**Figure S6** The estimated cost function.


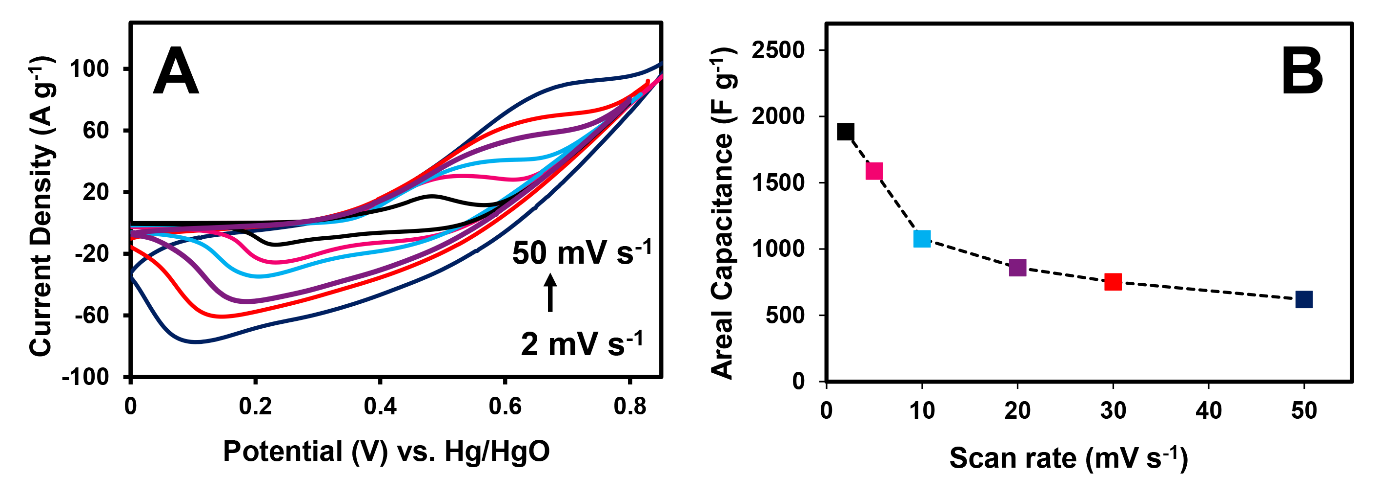


**Figure S7** **(A)** The NCH/PrGO/NF electrode's CV curves at different scan rates (2-50 mV/s) in KOH 1.0 M and **(B)** the relevance of the Areal capacitance of the NCH/PrGO/NF electrode with scan rate in KOH 1.0 M.


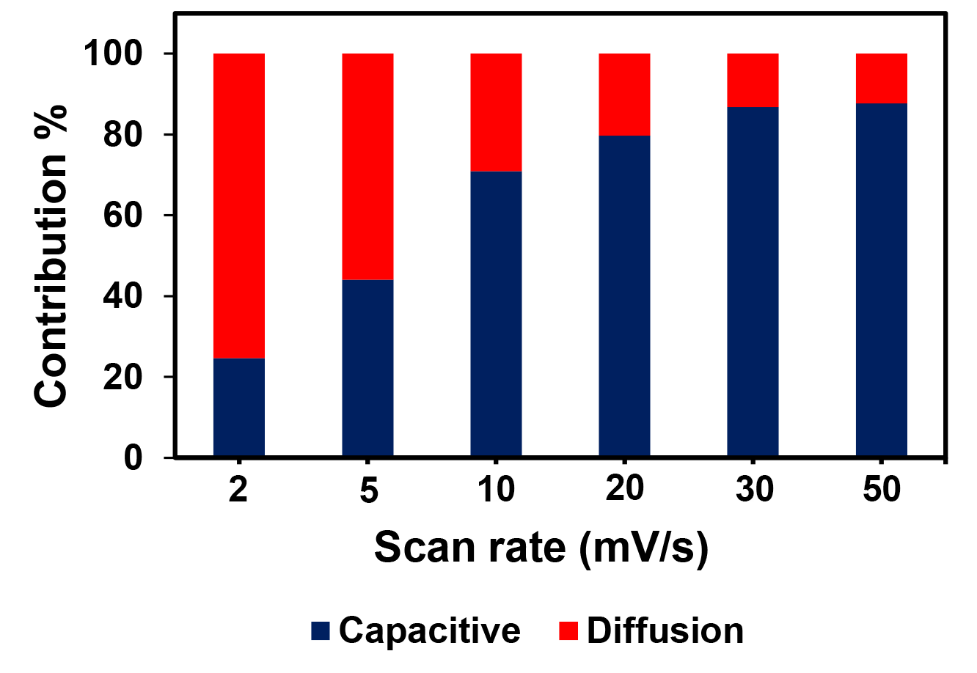


**Figure S8** **(A)** Capacitive and diffusion-controlled process contributions at different scan rates.


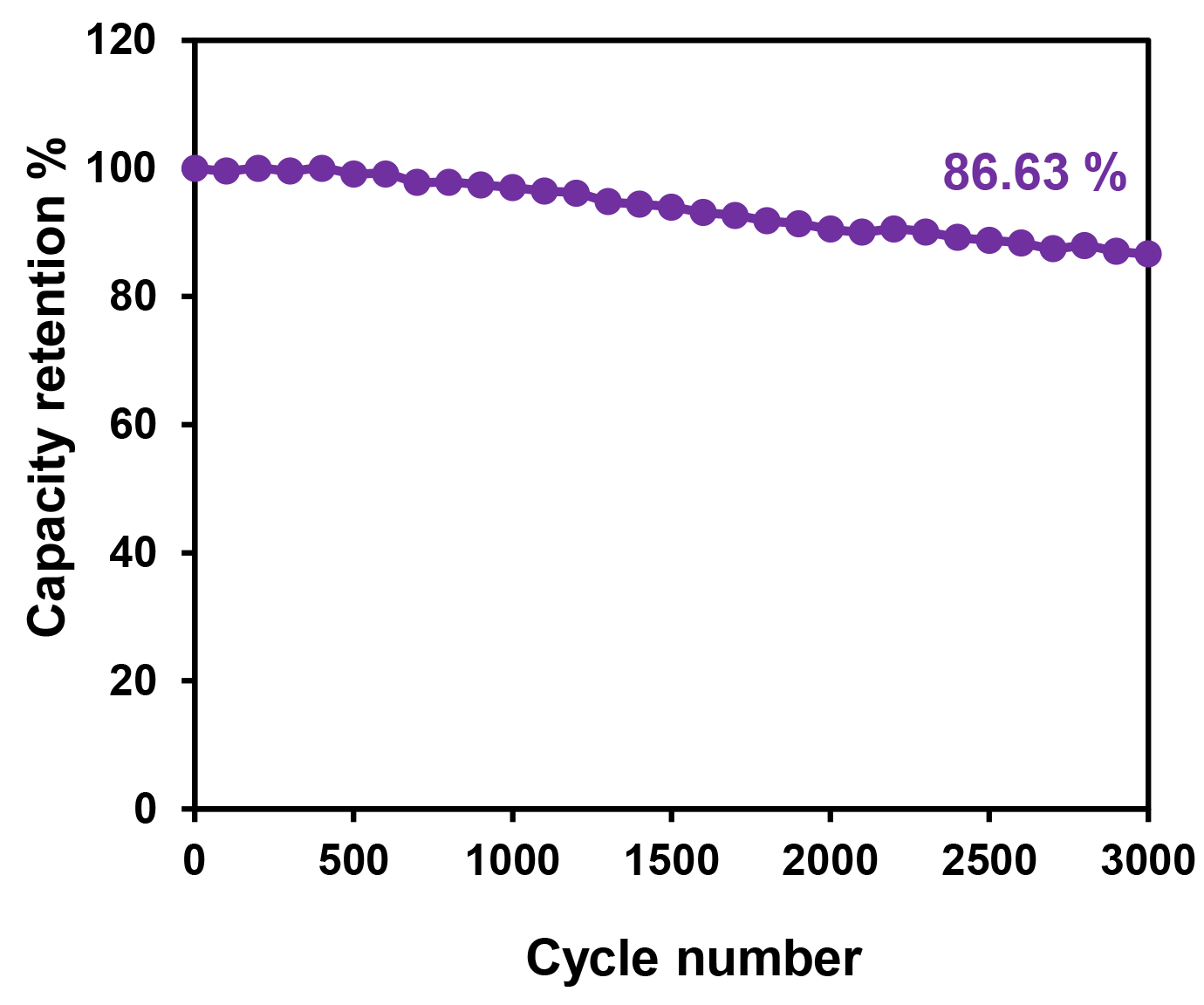


**Figure S9** The cycling performance of the NCH/PrGO/NF electrode at 10 A g^-1^ in the three-electrode system.


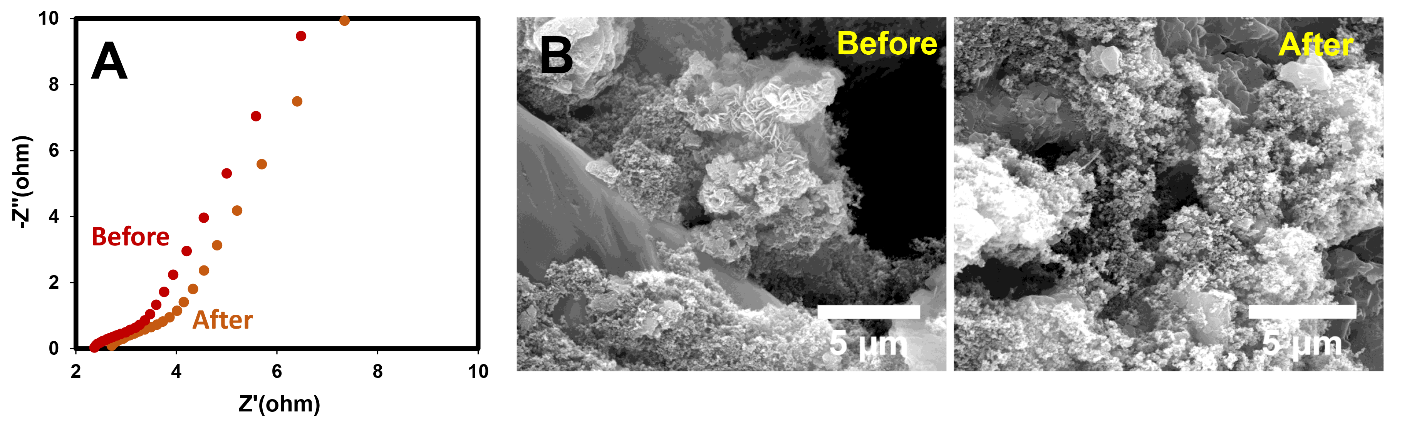


**Figure S10** **(A)** impedance spectra and **(B)** FE-SEM images of NCH/PrGO/NF electrode before and after cycling.


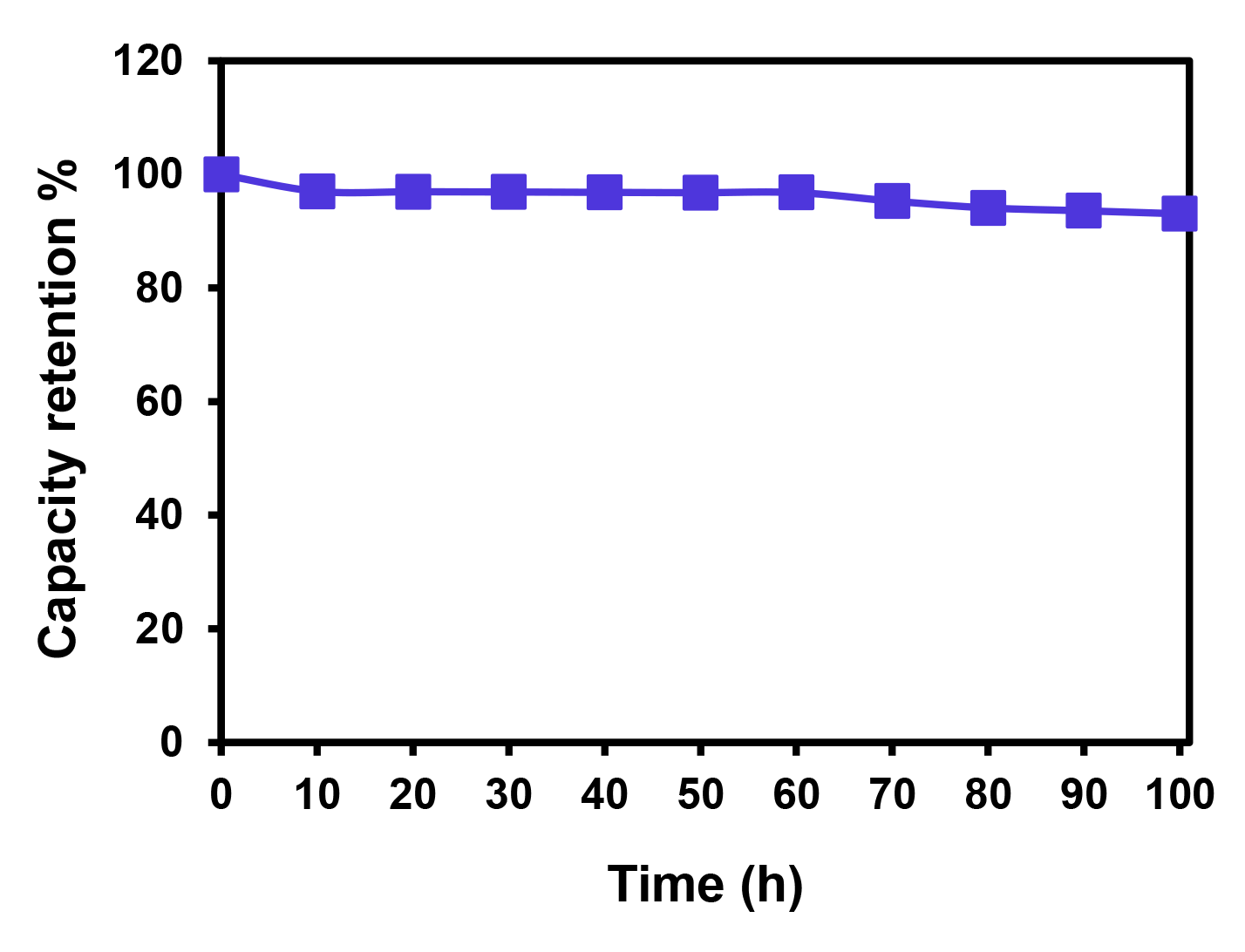


**Figure S11** Float voltage stability test of the NCH/PrGO/NF//AC/NF AHSC device for 100 h.

Experimental

Synthesis of PrGO Firstly, GO was synthesized by the modified hummer method.^1^ Afterward, a simple method was selected to prepare PrGO hydrogel based on previous literature^2^; in brief, 120 mg GO was dispersed into 60 mL of deionized water, then 0.1 g phytic acid (from rice grain) as a phosphorous source was added to it and ultrasonic for 3 h to obtain a uniform suspension. After that, this solution was transferred into a 100 mL Teflon-lined stainless-steel autoclave and kept at 170°C for 12 h, cooled naturally to room temperature, and washed with distilled water and ethanol (1:1) several times. Finally, the obtaining 3D PrGO hydrogel vacuum freeze-dried overnight for the next synthesis. (Figure S12)


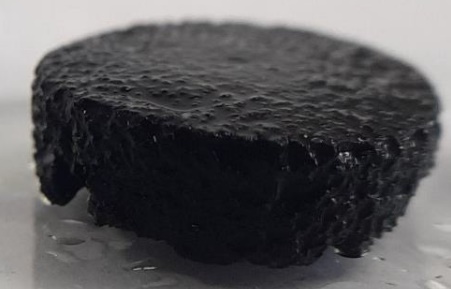


**Figure S12** Microscopic view of 3D PrGO hydrogel.

Apparatus The chemical structures of the materials were studied by X-ray photoelectron spectroscopy (XPS) (Bes Tec, Germany). The X-ray diffraction (XRD) patterns were obtained from an AW-XDM300 X-ray diffractometer (Asenware Co., China). Fourier transform infrared (FT-IR) spectra were studied with a 680-plus spectrometer (Jasco Co., Japan). The field emission-scanning electron microscope (FE-SEM) images were captured by scanning electron microscopy a MIRA3 (Tescan Co., Czech Republic) with an acceleration voltage at 15 kV equipped with map analysis and energy-dispersive X-ray analysis. Transmission electron microscopic (TEM) analysis was investigated with an Em-208s (Philips Co., Netherlands) and High-Resolution TEM (HR-TEM) carried out by a JEM-2100F (JEOL Co., Japan). The ICP-OES was analyzed via Vista-PRO (Varian, USA). UV-Vis spectra were investigated with a 680-plus spectrometer (Jasco Co., Japan). Brunauer - Emmet - Teller (BET) surface area analyzer was employed by NanoSord (IRAN).

Electrochemical measurements For preparing the working electrode, the 1*1 cm^2^ Nickel foam (NF) as the current collector was immersed in HCl (30 %) and 1:1 (v/v) solution of distilled water and ethanol to clean the surface of its for 5 mins, respectively. Then NF was dried in an oven (60°C) for a day. The slurry was prepared by mixing the active material, carbon black, and polyvinylidene difluoride (PVDF) (80:15:5 wt%) in N-methyl-2-pyrrolidone (NMP) and was loaded on NF (The mass loading of active material is about 5 mg). Afterward, the resulting electrode was dried in an oven (60°C) for 24 hrs. The electrochemical measurements of NCH/PrGO as an electrode material are studied for supercapacitor application in a three-electrode system, the Ag/AgCl electrode, platinum rod (2 mm diameter), and the prepared electrode was taken as reference electrode, a counter electrode, and working electrode in 1.0 M KOH aqueous solution, respectively.

Assembling and Electrochemical measurements of asymmetric supercapacitors (ASCs) The Asymmetric supercapacitor (ASC) devices were fabricated using NHC/PrGO/NF, activated carbon AC/NF, and a piece of *Whatman*® cellulose filter paper as the positive electrode, the negative electrode, and the separator, respectively. According to charge balance theory (q^+^ = q^-^), the optimal mass ratio of positive and negative electrodes
(m^+^ /m^-^) was optimized to enhance operating voltage and The ASCs device performance. The mass ratio of solid-state ASCs device is calculated by the equation (1):

(1)

$$\frac{m^{+}}{m^{-}}=\frac{C^{-}{\Delta V}^{-}}{C^{+}{\Delta V}^{+}}$$

*C*^-^ (F g^-1^) and *C*^+^ (F g^-1^) represent the specific capacitance of AC and NCH/PrGO/NF electrodes, respectively. Δ*V*^¯^(V) and Δ*V*^+^ (V) illustrate the potential window of AC and NCH/PrGO/NF electrodes, respectively.

The specific capacity (*C*) and capacitance (*Cs*) from the discharge curves, the energy density *E* (Wh kg^-1^), power density *P* (W kg^-1^), and the coulombic efficiency (*η*) of the ASC device were calculated by the equation (2-6):

$$C=\frac{I {\Delta t}_{d}}{m}$$

(2)

$$C_{S}=\frac{I {\Delta t}_{d}}{m \Delta V}$$

(4)

(3)

$$E=\frac{1}{2}C_{s}{\Delta V}^{2}$$

(5)

$$P=\frac{E}{{\Delta t}_{d}}$$

(6)

$$\eta=\frac{{\Delta t}_{d}}{{\Delta t}_{c}}$$

*I* (A) represent the discharge current, *m* (g) represents the total mass of active materials on electrodes, Δ*V* shows operating voltage (V), Δ*t_d_* (s) represents the discharge time, and Δ*t*_c_ represents the charging time.

All the electrochemical measurements were carried out on a Bio-Logic (SP-300) system, and EC-Lab software was also used to analyze electrochemical curves.

**References**

1. Ghiaci, M., Farahmand, S., Masoomi, K., Safaiee, M. & Razavizadeh, J. S. Graphene vanadic acid (GVA) as a novel heterogeneous catalyst for highly selective benzene hydroxylation under mild conditions. *Adv. Powder Technol.* **32**, 660–669 (2021).

2. Fan, X. *et al.* Preparation and supercapacitive properties of phosphorus-doped reduced graphene oxide hydrogel. *Electrochim. Acta* **330**, 135207 (2020).
